# Abnormal B Cell Development in Systemic Lupus Erythematosus: What the Genetics Tell Us

**DOI:** 10.1002/art.40396

**Published:** 2018-02-22

**Authors:** Sarah Karrar, Deborah S. Cunninghame Graham

**Affiliations:** ^1^ King's College London London UK

## Introduction

Systemic lupus erythematosus (SLE) is an autoimmune disease characterized by B cell dysfunction, production of autoantibodies directed toward cellular and nuclear components, and multiorgan damage caused by immune complex deposition and inflammation within affected tissues 1. It largely affects women of childbearing age (the third and fourth decades of life) and is associated with significant morbidity and mortality.

In healthy individuals, B cells with autoreactive receptors are selected out during B cell maturation, starting at the initial stages of B cell receptor (BCR) development in the bone marrow and continuing through to the fine tuning that occurs in activated mature B cells in secondary lymphoid tissue. Studies in lupus patients as well as mouse models indicate that these processes are altered in SLE.

The etiology of the disease is complex and its phenotype is highly heterogeneous, but genetic susceptibility is thought to contribute as much as 60% of disease risk 2. Although rare monogenic causes do exist, heredity in SLE is complex, with multiple common variants contributing to disease, with patients having to achieve a certain “genetic threshold” for disease risk. This genetic risk, in combination with environmental factors (exposure to ultraviolet sunlight, smoking, and infections including Epstein‐Barr virus have all been implicated), leads to development of the disease 1. In this review, we summarize some of the B cell anomalies in SLE and incorporate evidence from studies in humans and mouse models, together with data from genetic association studies, to explain the mechanisms behind B cell dysregulation in SLE.

## The B cell phenotype in SLE

The crucial role of B cells in SLE pathogenesis is well recognized, from producing autoantibodies to abnormal regulation of immune responses 3, 4. Various abnormalities have been noted in SLE B cells. First, there is an imbalance of B cell subtype numbers, with an increase in class‐switched memory B cells relative to naive B cells 3. Second, B cells from SLE patients have exaggerated BCR responses, with receptor crosslinking leading to increased calcium influx and tyrosine phosphorylation of downstream signaling molecules 3. Increased memory B cell numbers confer significant disease risk as these have a lower activation threshold, allowing autoreactive B cells to thrive with minimal antigen contact, while enhanced receptor activation contributes to the steady‐state active phenotype seen in SLE 3, 5.

B cells contribute to disease mainly by producing autoantibodies targeting nuclear components including DNA (anti–double‐stranded DNA [anti‐dsDNA]), RNP particles (anti‐Ro, anti‐La, and anti‐Sm), histones, and nonhistone chromatin proteins. These are present in >90% of patients and contribute to disease progression via immune complex formation 6. Titers of these autoantibodies (especially anti‐dsDNA) correlate positively with increased disease activity, and serial measurements are used to monitor patients for disease flares 6. There is also evidence that autoantibodies cross‐react with cellular components other than nuclear targets 7. For example, anti‐dsDNA antibodies bind to major glycosaminoglycan components in the glomerular basement membrane, suggesting a possible direct role in nephritis 7. In mouse models, transfer of autoantibodies from diseased to unaffected animals leads to development of typical immune complex–mediated nephritis 8. Moreover, in MRL/*lpr* mice (which develop lupus‐like disease spontaneously), disease severity can be attenuated and mortality reduced by ~50% if antibody secretion is blocked, providing robust evidence that autoantibodies are more than spectators in disease etiology 9.

A recent explosion in genome‐wide association studies (GWAS) has identified >80 potential risk loci across multiple immunopathologic pathways 10. In this review, we discuss how genetic variants affect the development of B cells, allowing them to overcome several checkpoints to break self tolerance, and how they contribute to the abnormal active phenotype observed in SLE. We examine how these genes alter both early developmental pathways in the bone marrow and late maturation processes to cause B cell dysregulation.

## Central tolerance checkpoint of B cell development in the bone marrow in SLE

**Figure 1:**
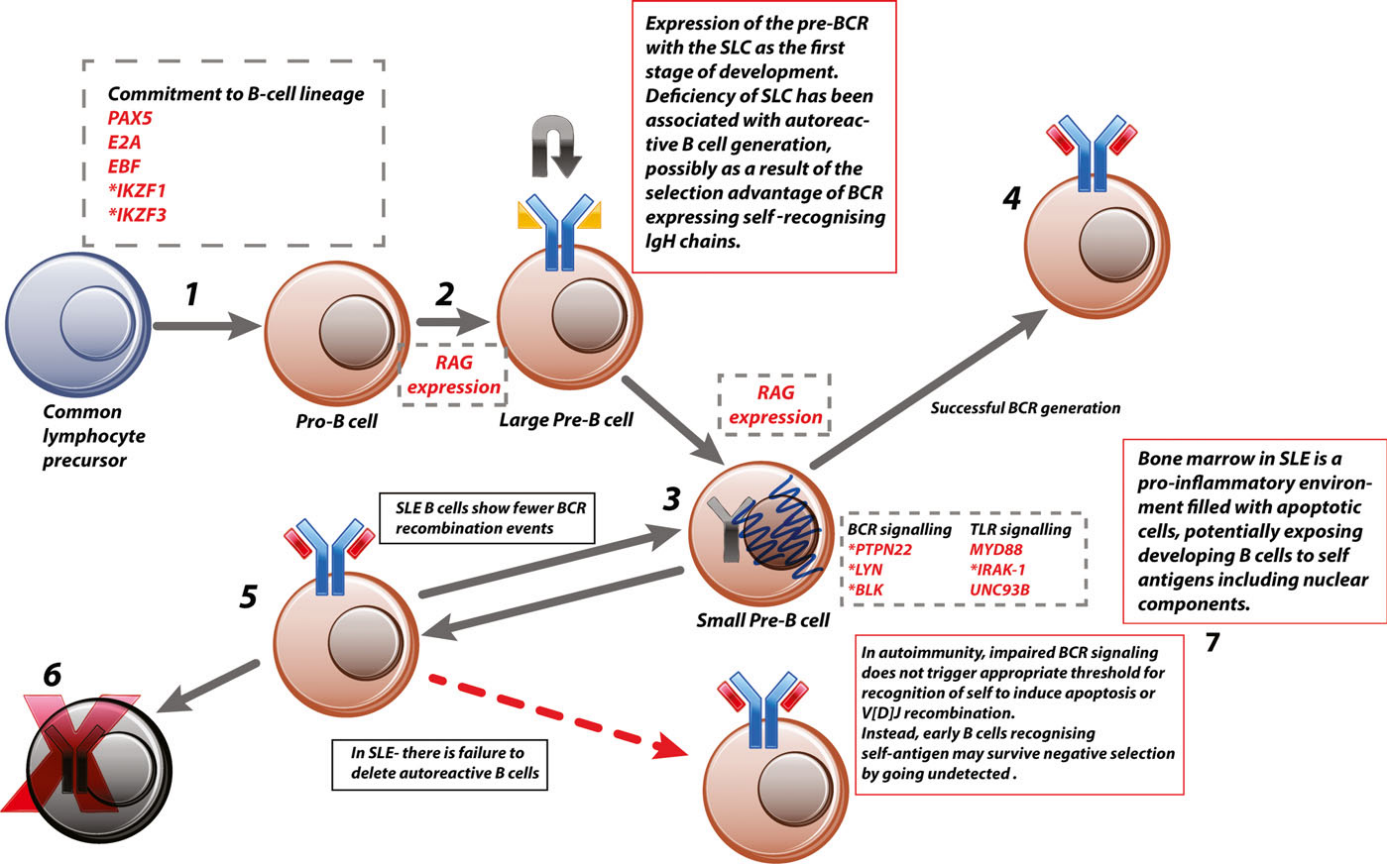
Central tolerance. **1,** Common lymphocyte precursor commits to B cell lineage via expression of B cell–specific transcription factors (e.g., early B cell factor [EBF]), which initiates IgH rearrangement. **2,** Expression of the generated IgH component of the pre–B cell receptor (pre‐BCR) is combined with the surrogate light chain (SLC). **3,** Successful signaling through the pre‐BCR leads to a short burst of proliferation and internalization of the pre‐BCR and commences a second wave of recombination, this time in the light‐chain gene. **4,** The generated BCR is then assessed for self‐recognition. Those cells that have generated non–self‐recognizing BCRs with functioning signaling switch off recombination‐activating gene (RAG) expression and become immature B cells. **5,** Because V[D]J recombination is a stochastic process, a proportion of pre–B cells will generate autoreactive BCRs. This is detected by excess BCR signaling due to high‐affinity binding within the bone marrow or abundance of antigen. This leads to continued V[D]J recombination until acceptable BCR is generated or all possible recombination has been exhausted. **6,** Failure to generate a non–self‐recognizing BCR leads to apoptosis. **7,** In autoimmune disease this process is impaired, potentially by reduced signaling through the developing BCR, which fails to trigger the threshold for apoptosis. Genes or proteins involved at each stage are shown in dashed boxes. * = genes identified as risk‐associated loci in systemic lupus erythematosus (SLE). TLR = Toll‐like receptor; MyD88 = myeloid differentiation factor 88; IRAK‐1 = interleukin‐1 receptor–associated kinase 1; Unc‐93B = Unc‐93 homolog B.

Normal B cell development starts in the bone marrow, where the first round of negative selection of autoreactive B cells (termed central tolerance) occurs. This process is summarized in Figure 1. Many potential abnormalities in central tolerance have been implicated in SLE, including failure of adequate negative selection of autoreactive B cells and inadequate receptor editing (steps 6 and 3, respectively, in Figure 1), both of which are critical steps in maintaining tolerance to self 11.

The molecular mechanisms by which SLE autoreactive B cells evade central tolerance have yet to be fully elucidated. There are some clues, however, from various genetic studies 12. Patients with single‐gene mutations resulting in primary immunodeficiencies frequently develop a wide range of autoimmune diseases in addition to increased susceptibility to infections 12. Their mutations teach us that central tolerance is largely dependent on adequate BCR signaling in the bone marrow 12 (steps 2 and 3 in Figure 1). In X‐linked agammaglobulinemia, a defect in the gene for Bruton's tyrosine kinase (needed for downstream BCR signaling) results in increased frequency of autoreactive B cells 12. One possible mechanism is that binding to self antigen does not induce a strong enough response in BCR signaling to trigger clonal deletion 12. Conversely, deficiency in Wiscott‐Aldrich syndrome protein (WASP; a negative regulator of BCR signaling) results in more stringent central control mechanisms, with WASP‐knockout mice showing a much lower proportion of autoreactive B cells being released from the bone marrow. Their mature B cells also show abnormal peripheral tolerance and hyperactive phenotype, possibly driven by T cell abnormalities 12.

Outside of single‐gene mutations, GWAS have expanded our knowledge of the molecular basis of B cell developmental anomalies in SLE 13, 14. These GWAS have identified several SLE susceptibility loci near genes known to be important for early B cell development and BCR signaling (see Table 1 for summary). Variants affecting BCR signaling are discussed in more detail later in this review 13, 14.

**Table 1:** Summary of loci identified in genome‐wide association studies and their role in B cell development^a^

Gene	Role in B cell development	Effect of risk variant	Mechanism of contribution to disease	B cell phenotype
Early B cell development and central tolerance				
*IKZF1*	Transcription factor helps regulate transition of multipotent progenitor cells to pro–B cells and pre–B cells	Regulates *IKZF1* expression, possibly increasing expression; also affects expression of C1qB and several IFN response genes 11	Unknown	Knockout mice show developmental block in early B cell differentiation 13
*IKZF3* b	Transcription factor helps regulate transition of pro–B cells and pre–B cells; also important in differentiation of plasma cells and development of B cell memory	Unknown	Unknown	Block in early B cell differentiation at the pre–B cell stage 13; knockout mice lack B cell immunologic memory and develop SLE‐like disease 48
B cell maturation in lymphoid tissue and peripheral tolerance				
*ETS1*	Transcription factor regulating B cell development in the GC	Down‐regulation of expression 25	Risk allele increased binding of pSTAT‐1, leading to reduced expression 25	Transgenic knockout mice show inability to induce B cell anergy in autoreactive cells 25
*UBE2L3*	Ubiquitin‐conjugating enzyme E2, which modulates NF‐κB activity	Increased expression in B cells 26	Increases NF‐κB activation via LUBAC‐mediated ubiquitination of IκBα 26	Higher numbers of plasmablasts and plasma cells; basal active state in unstimulated B cells; modulated response to stimulatory signals such as TNF 26
*MHCII*	Antigen‐presenting molecules expressed on activated B cells	Variants in HLA–DRB1, DQA1, and DQA2 have all been reported; effect of risk variants unknown	Potentially enhanced presentation of autoantibodies in disease	HLA–DRB1*03:01 variant associated with anti‐La and anti‐Ro (71)
*CD80*	Costimulatory molecule expressed on activated B cells and promotes T cell activation via engagement with CD28 on T cell membrane	Unknown	Unknown	CD80 is known to be overexpressed in B cells from SLE patients (72)
BCR and pre‐BCR signaling molecules affecting both central and peripheral tolerance				
*PTPN22*	Pre‐BCR and BCR signaling molecule	Loss of function of *PTPN22* due to missense mutation 28, 29	Altered central tolerance with no clonal deletion of autoreactive B cells 28, 29; peripheral B cells show enhanced activation via CD40 28, 29	Subjects with the risk variant have impaired BCR signaling, more autoreactive B cells 28, 29; active peripheral B cells 28, 29
*BLK*	Src family kinase important in pre‐BCR signaling	Down‐regulation of Blk expression 33; splice variant which is prone to inactivation and degradation 34	Potentially reduced pre‐BCR signaling and failure of central tolerance 33	Blk‐knockout mice develop autoimmune disease similar to SLE and increase in B‐1a cells; humans with the risk phenotype also showed increased levels of anti‐dsDNA (even healthy individuals) 33, 34
*CSK*	Src family kinase important in BCR signaling	Increased expression of Csk 30	Increased Lyp phosphorylation and augmented BCR signaling 30	Active mature B cell phenotype 30
*LYN*	Src family kinase important in pre‐BCR signaling	Unknown	Unknown; variant is protective against severe disease 31	SLE patients demonstrate reduced expression; knockout mice develop lupus‐like disease with aberrant BCR signaling 31
*TLR7*	Toll‐like receptor	Increased TLR‐7 expression 11; increased IFN response genes 11	Possible enhanced IFN signaling 11; possible altered handling of nuclear material, increasing risk of anti‐DNA antibodies (40)	Patients with the variant are at increased risk of nephritis and more likely to have anti‐dsDNA antibodies 40
Memory cells and long‐lived plasma cells				
*OX40L*	Membrane‐bound protein on memory B cells; member of TNFSF important in GC T cell–B cell interaction	Unknown	May augment memory B cell and T cell stimulation, leading to more active B cell phenotype 46	Possible active memory B cell phenotype; OX40L levels correlate with disease activity and more severe disease 46
*BACH2*	Transcription factor which regulates GCs; B cell differentiation into memory B cells and class‐switching; role in central tolerance checkpoint and pre‐BCR signaling 47	Unknown	Unknown	Knockout mice have reduced B cell numbers and reduced numbers of memory B cells (47)
*PRDM1*	Encodes for BLIMP‐1, a transcription factor important for plasma cell differentiation; negatively regulated by ETS‐1; repressor of IFNγ gene expression	Reduced expression in DCs 55; no effect in B cells 55; unknown effect in plasma cells	Possible active DC phenotype promoting B cell stimulation and differentiation (55)	Conditional DC‐knockout female mice develop SLE and increased numbers of GC B cells (55)
*BANK1*	Scaffolding protein involved in BCR signaling	Splice variant 49, 50	Variant forms larger and more widespread scaffold, potentially augmenting BCR signaling 49, 50	Altered BCR‐ and CD40‐mediated signaling, expansion of memory B cell numbers (49,50)

a Where possible, the effect of the risk variant is included along with the way it potentially contributes to the B cell phenotype in systemic lupus erythematosus (SLE). IFN = interferon; GC = germinal center; LUBAC = linear ubiquitin chain assembly complex; TNF = tumor necrosis factor; BCR = B cell receptor; anti‐dsDNA = anti–double‐stranded DNA; TLR‐7 = Toll‐like receptor 7; TNFSF = TNF ligand superfamily; BLIMP‐1 = B lymphocyte–induced maturation protein 1; DCs = dendritic cells.

b Also has crucial role in long‐lived plasma cell development.

In early B cell development, several stages have been described which are associated with distinct genetic and molecular events. Two of these genes have been identified as risk loci and are discussed in the next section.

## SLE risk loci and their role in the commitment to B cell lineage, common lymphoid progenitors, B lymphocyte precursors, pro–B cells, and pre–B cells

Commitment of the multipotent progenitor cells in the bone marrow to lymphocyte development depends on the expression of several transcription factors, including Ikaros (encoded by *IKZF1*) and Aiolos (encoded by *IKZF3*), among others 13, 15 (step 1 in Figure 1). Ikaros in particular is known to be crucial for early commitment to B cell lineage. Its exact role in the early multipotent progenitor cells is unknown, although we know that mice deficient in Ikaros fail to develop any common lymphocyte precursors, with arrest of B cell development before lineage commitment to the B cell–biased lymphoid progenitor 16. Low expression of Ikaros allows generation of some B cells, but overall numbers remain low and differentiation is impaired at all stages 16.

The pre–B cell stage is characterized by the expression of the pre‐BCR, and successful signaling through the pre‐BCR arrests recombination of the IgH chain and the initiation of expression of the Ig light chains of the final BCR 17 (step 2 in Figure 1). Ikaros and its closely related family member Aiolos are both induced on engagement of the pre‐BCR and help terminate signaling through the pre‐BCR (steps 2 and 3 in Figure 1), promote exit from the cell cycle, and allow rearrangement of the Ig light‐chain genes. Ikaros also induces expression of recombination‐activating gene 1 (RAG‐1) and RAG‐2 and is required for IgH V_H_ gene recombination, allowing the pro–B cell to progress to the large pre–B cell stage (18).

Both the Ikaros and Aiolos variants (single‐nucleotide polymorphism [SNP] rs4917014, which lies within the 3′‐untranslated region [3′‐UTR] of the *IKZF1* gene [encoding Ikaros] [*P* = 2.7 × 10^–23^], and SNP rs2941509, which lies within the 5′‐UTR of the *IKZF3* gene [encoding Aiolos] [*P* = 3.198 × 10^−6^]) have been associated with increased transcription of their respective genes in whole blood 13, 15 (Table 1). In models of overexpression in pre–B cell lines, increases in Ikaros and/or Aiolos induce termination of IgH recombination and stop signaling through pre‐BCRs. However, high levels of expression are required to induce this process; therefore, it is plausible that both of these variants are promoting early transition to the small pre–B cell and may be contributing to the inadequate receptor editing observed in SLE 11, 16, 19.

Humans with germline mutations in *IKZF1* have an early block in common lymphocyte precursor development, with reduced pro–B cell numbers and normal pre–B cell numbers 20. Approximately half of the reported patients also developed autoimmune disease, including 1 who had SLE, suggesting that dysfunction and abnormalities of early B cell development can result in both immunodeficiency and autoimmunity 20.

## Checkpoint at B cell maturation in the lymphoid tissue, from immature B cell to plasma cell and peripheral tolerance

**Figure 2:**
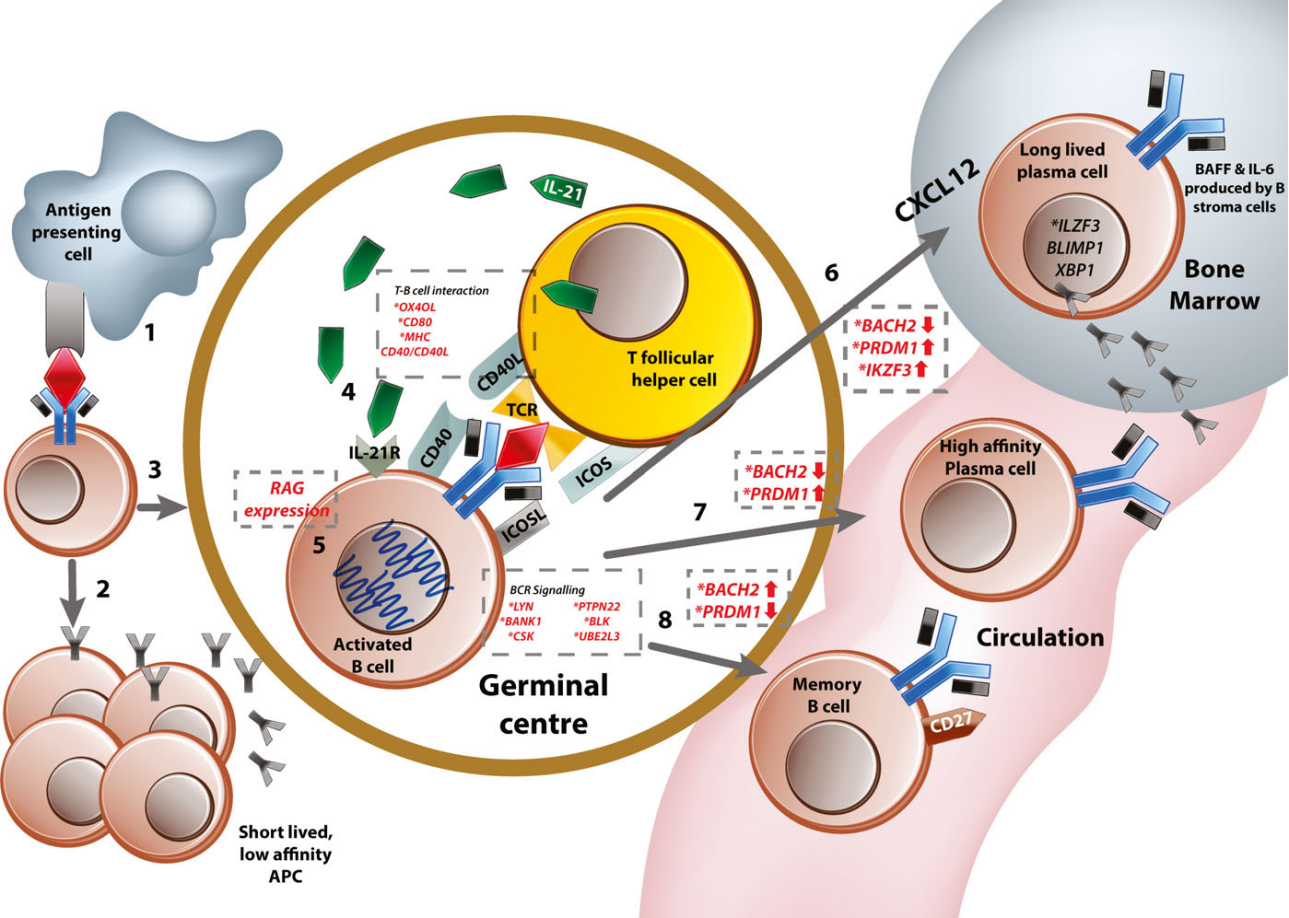
Peripheral tolerance. **1,** Naive B cells in the marginal zone encounter their relevant antigen as presented by resident antigen‐presenting cells (APCs). **2,** Some activated B cells remain outside the germinal center and become short‐lived low‐affinity antibody‐producing cells. **3,** Activated B cells migrate to the germinal center (under influence of CXCL12 produced by bone marrow stromal cells), **4,** where they interact with follicular helper T cells whose T cell receptors (TCRs) recognize self antigen. This also involves bidirectional signaling through multiple costimulatory molecules and the B cell receptor (BCR). **5,** At this stage, most B cells undergo a round of somatic hypermutation to achieve affinity maturation. This requires expression of the *RAG* genes. Following this, activated B cells can differentiate into 3 potential cell types. **6,** Long‐lived plasma cells are selected from the pool of B cells with the highest affinity receptors. They up‐regulate expression of CXCR4 and migrate toward their niche (usually in the bone marrow), where they reside and continue to produce background antibody. **7,** Some activated cells terminally differentiate into high‐affinity plasma cells, which are responsible for the “second wave” of high‐affinity antibody after antigen exposure. **8,** B cells with low‐affinity BCRs are preferentially selected to become memory B cells. Genes or proteins involved at each of the regulatory stages are shown in dashed boxes. * = genes identified as risk‐associated loci in systemic lupus erythematosus (SLE). IL‐21 = interleukin‐21; MHC = major histocompatibility complex; IL‐21R = IL‐21 receptor; RAG = recombination‐activating gene; ICOS = inducible costimulator; BANK‐1 = B cell scaffold protein with ankyrin repeats 1; UBE2L3 = ubiquitin‐conjugating enzyme E2 L3; BLIMP‐1 = B lymphocyte–induced maturation protein 1; XBP‐1 = X‐box binding protein 1.

Many abnormalities in peripheral tolerance have been identified in SLE, from problems with somatic hypermutation to memory B cell dysfunction (see Figure 2 for summary). First, SLE patients show aberrant and raised RAG expression in peripheral B cells (step 6 in Figure 2), raising the possibility that some autoreactive B cells arise as a result of mutation of a “healthy” BCR into one that recognizes self antigen 21. This hypothesis is supported by analysis of genetic variation in the Ig produced by autoimmune mice, showing that point mutations can render a previously self‐tolerant BCR autoreactive 22. These data have been replicated in the analysis of anti‐dsDNA antibodies from humans with SLE 23. Second, autoantibodies in SLE often evolve over the duration of the disease, recognizing different epitopes of their respective antigens and frequently achieving a higher affinity 23, 24. The raised levels of interleukin‐6 (IL‐6) seen in the disease may contribute to this, as IL‐6 is a known up‐regulator of RAG gene expression 25.

The other major player in this process of secondary maturation and in the maintenance of tolerance is the T cell–B cell interaction (step 5 in Figure 2). This is supported by evidence from single‐gene immunodeficiency disorders, mouse models, and GWAS 12, 14, 26. Patients with single‐gene mutations in CD40, CD40L, and major histocompatibility complex (MHC) class II develop a significant proportion of autoreactive B cells, including those with receptors recognizing nuclear components (including antinuclear antibodies [ANAs]) 26. This is despite their having normal central tolerance processes in the bone marrow 12, 26. In the *Sle1* murine model, disease risk is inherited via a region on chromosome 1, carrying polymorphisms in genes encoding multiple receptors needed for T cell–B cell interactions (including *Slam*,* Ly108*,* Cd84*,* Cracc*, and *Ly9*) 27. In the germinal centers (GCs) of these mice, transient short‐term contact between B cells and T cells allows rare autoreactive B cells and T cells to “sample” many different cells in the GC, increasing chances of interaction for positive costimulation 27. Shorter contact times between immune cells are also known to alter their function (e.g., poorer Treg cell ability to induce tolerance in target cells due to shorter contact times) 28. It is possible that shorter T cell–B cell contact in the GC may lead to chronic, low‐grade activation, allowing autoreactive B cells to survive due to background low‐quality contact, without receiving adequate signals to become anergic.

Patients and mice with deficiencies in CD40, CD40L, and MHC class II who have poor T cell–B cell interaction overcome lack of B cell stimulation by up‐regulating BAFF, a stimulatory cytokine that promotes B cell survival and proliferation 26. This has important consequences for B cell activation and murine models in which mice that overexpress BAFF develop lupus‐like disease with ANAs and anti‐dsDNA 29. These findings suggest that in the absence of specific and controlled BCR activation, more generic signals (such as BAFF) promote indiscriminate B cell activation and survival of self antigens recognizing B cells and normal foreign antigens recognizing cells equally (29). Variants in loci near or within *OX40L*/*TNFSF4,* MHC class II, and *CD80* have all been implicated as associated with risk of SLE in various GWAS 13, 14, 30, 31 (Table 1).

The SLE‐associated variant rs6590330 in the promoter of the gene *ETS1* results in down‐regulation of expression of the transcription factor ETS‐1 in whole blood from humans 30. Studies in transgenic mice that are both deficient in Ets‐1 and express an autoreactive BCR indicate that while central tolerance is maintained, deficiency in Ets‐1 results in impaired anergic responses 32. B cells from Ets‐1^–/–^ mice produced autoantibodies despite receiving appropriate “anergy” signals and continued to secrete them even in the absence of interaction with cognate antigen 32. Some features of B cell anergy (such as reduced BCR‐mediated tyrosine phosphorylation and Ca^2+^ influx) could be induced in deficient cells after stimulation with high‐affinity antigen; however, this could not switch off autoantibody production 32.

The *UBE2L3* gene (which codes for ubiquitin‐conjugating enzyme E2, also known as UBCH7) on chromosome 22 contains a risk variant (associated SNP rs140490, *P* = 7.5 × 10^–8^) within its promoter that leads to increased expression and protein levels in B cells (but not in other immune cells such as T cells) 31. UBCH7 drives NF‐κB activation through increased linear ubiquitin chain assembly complex–mediated ubiquitination and subsequent proteasomal degradation of IκBα, an inhibitor of NF‐κB 31. Raised basal levels of NF‐κB led to steady‐state activation and augmented responses to stimulatory signals 31. This resulted in higher numbers of both plasmablasts and plasma cells and modulated response to stimulatory signals such as CD40 and tumor necrosis factor (TNF) (Table 1 and step 5 in Figure 2) in carriers of the risk variant 31.

## Variants affecting B cell signaling molecules affect both peripheral and central tolerance mechanisms

Variants affecting B cell and pre–B cell signaling affect both central and peripheral tolerance, and many genes involved in both BCR and pre‐BCR signaling have been identified from GWAS 13, 14. These variants contribute to the generation of autoreactive B cells and the activated phenotype identified in peripheral B cells in SLE 13, 14 (Table 1). Variants resulting in impaired BCR signaling are thought to contribute to autoimmunity in a mechanism analogous to that noted in the monogenic immunodeficiencies discussed above, in which inadequate pre‐BCR signaling leads to failure in reaching the threshold for clonal deletion or BCR rearrangement 12, 33. Variants contributing to increased BCR‐mediated signaling may promote the peripheral active B cell phenotype characteristic of SLE 34, 35. Risk variants identified in SLE include PTPN22, Blk, Csk, and Lyn 13, 14, 36 (step 3 in Figure 1 and step 4 in Figure 2).

The risk allele (SNP rs476601, *P* = 3.4 × 10^–12^) for *PTPN22*, which encodes the tyrosine kinase Lyp, is associated with impaired BCR signaling and is thought to contribute to autoreactive B cell survival through various mechanisms 14 (Table 1). This risk variant is associated with faulty central tolerance and failure to remove autoreactive B cells in the bone marrow 33. In addition, peripheral B cells carrying this risk allele up‐regulated many genes involved in B cell activation, including those involved in BCR, CD40, and Toll‐like receptor (TLR) signaling as well as cytokine receptors (e.g., IL‐21 receptor [IL‐21R] and IL‐4R) 34. Moreover, these B cells show increased surface expression of CD40 as well as enhanced responses to CD40 engagement, potentially contributing to the active peripheral phenotype 34. Up‐regulation of these proactivation genes is thought to be a mechanism by which B cells overcome the deficit in Lyp function caused by the risk variant 34.

Blk, a Src family tyrosine kinase, is normally up‐regulated at the pre–B cell stage and is important in the transduction of pre‐BCR signaling 37. Blk phosphorylates the Ig α‐ and β‐subunits of the BCR and is known to bind the phosphatidylinositol lipase C‐γ230, forming a complex with the B cell adaptor protein ankyrin repeats (encoded by the *BANK1* gene and, interestingly, also a susceptibility locus for SLE) during BCR signaling 38. The disease‐associated SNPs for Blk (rs922483 and rs1382568) both result in the down‐regulation of Blk expression and potential failure of adequate pre‐BCR signaling 36, 39 (Table 1). A third and rarer coding variant of Blk associated with SLE that results in an alanine‐for‐threonine substitution in its SH3 domain leads to hypophosphorylation, inactivation, and rapid degradation of the protein 40. This variant also demonstrates impaired binding to B cell scaffold protein with ankyrin repeats 1 (BANK‐1) scaffolding, which is important in signal transduction 40.

Blk^–/–^ mice develop autoimmune disease, with anti‐dsDNA autoantibodies and immune complex–mediated glomerulonephritis, together with an increase in the proportion of B‐1 cells (a low‐affinity IgM‐producing subtype of B cells in mice) 41. Expansion of this subtype of B cells in other murine models of lupus is well documented and is thought to be important, particularly in driving nephritis 41. In a mechanism similar to that of the Lyp variant, this may contribute to autoimmunity by impairing central tolerance mechanisms. However, abnormalities in peripheral BCR signaling have also been described, with low baseline BCR activity at rest but enhanced responses on activation, heightened ability to stimulate T cells, and increased numbers of isotype‐switched memory B cells 42.

*CSK*, located on chromosome 15, carries a risk variant (SNP rs34933034) that up‐regulates expression of Csk, a Src family tyrosine kinase important in the BCR signaling cascade 35. B cells from those carrying the risk allele show increased levels of Csk expression and increased subsequent phosphorylation of Lyp, augmented BCR responses, and activation of mature B cells 35.

Lyn, another member of the Src kinase family, has also been associated with SLE 36, 43. Initial GWAS identified rs57829816 in the 5′ region (*P* = 5.4 × 10^–9^) and rs2667978 in the 3′ region (*P* = 5.1 × 10^–8^) in women of European ancestry, with both variants being protective. Further transancestral case–control studies identified a third variant in American women of European ancestry, rs6983130 in the 5′‐UTR of the gene (*P* = 0.000111) 36. This variant was also protective and was associated with less severe disease within cases 36. No association between any Lyn variants and disease has been identified in populations of African or Asian ancestry, nor has there been any association with changes in protein or transcript levels 36, 43, 44. However, data from SLE patients have shown them to have lower levels of Lyn compared to healthy controls, implying that lower levels may be associated with disease 45, 46. In addition, studies in B cell lines show that Lyn is required for inhibitory signals in response to weak BCR crosslinking 47. Lyn^–/–^ mice are susceptible to an inducible immune complex–mediated nephritis as well as to antibody‐induced arthritis 47.

Patients deficient in IL‐1R–associated kinase 4, myeloid differentiation factor 88, and Unc‐93 homolog B (signaling molecules in the TLR pathway) also have high levels of autoreactive B cells, suggesting that impaired signaling via these pathways results in survival of autoreactive BCRs, possibly by failing to pass the signaling threshold for clonal deletion in the bone marrow 12 (step 3 in Figure 1). These patients also demonstrated impaired receptor editing and had defects in peripheral tolerance 48. SNP rs3853839 in the 3′‐UTR of TLR‐7 (*P* = 2 × 10^–9^) has been associated with SLE in several GWAS 14. This variant is associated with increased transcript levels and amplified signaling, resulting in an enhanced interferon (IFN) signature 14. A study in Danish patients with SLE found that variants in TLRs 7–9 were associated with certain disease phenotypes and specific serologic markers 49. For example, some TLR‐7 variants were associated with nephritis and anti‐dsDNA antibodies 49.

## Checkpoint at the level of memory cells and long‐lived plasma cells in SLE

Although abnormalities in both central and peripheral tolerance are pivotal to pathology, memory B cells and long‐lived plasma cells contribute substantially to disease propagation 50. One of the most striking B cell findings in SLE is the high prevalence of mature, antigen‐experienced class‐switched memory cells 3. The effect of having a relatively large proportion of these cells is multifold. First, these cells are primed for action and have a lower activation threshold compared to naive B cells. Second, they exhibit resistance to regulatory and inhibitory signals 3. In addition, SLE patients have a unique group of memory B cells in the periphery which lack CD27 expression (a hallmark of memory B cell phenotype), the numbers of which correlate with increased disease activity, renal involvement, and higher serum levels of autoantibodies 51.

The endurance of autoreactive long‐lived plasma cells and their resistance to treatments such as B cell depletion and chemotherapy demonstrate the importance of this plasma cell subtype in disease persistence. In lupus‐prone mice, long‐lived plasma cells develop early and continue to expand throughout their lifetime 50. Murine models indicate a prominent role for these long‐lived plasma cells in autoimmunity; for example, antibodies derived from long‐lived plasma cells were found in diseased kidneys of NZB/NZW mice 8. In addition, these long‐lived plasma cells are thought to be responsible for the production of anti‐RNA antibodies, which explains why the titers of these antibodies are not altered with B cell depletion therapy 52, 53.

Development and survival of long‐lived plasma cells and memory cells is complex and depends on a functioning GC 54. Within the GC, there is stringent selection of cells with high‐affinity receptors to become long‐lived plasma cells, while memory B cells undergo a less rigorous selection process (Figure 2) 54. Memory B cells do acquire increased affinity over time, and some studies suggest that they may re‐enter the follicle and undergo further maturation. While long‐lived plasma cells reside in “niches” usually within the bone marrow, memory B cells are found circulating in the periphery and dwelling in secondary lymphoid tissue 54.

Various GWAS in SLE have identified an association with the gene encoding the protein OX40L/TNF ligand superfamily member 4 55 (Table 1 and step 5 in Figure 2). This gene codes for a membrane‐bound protein expressed on the surface of memory B cells, with its unique receptor OX40 primarily expressed on CD4+ Th cells. The OX40L–OX40 interaction results in bidirectional signaling in both the B cell and T cell involved, activation in both cells, and augmentation of the immune response 55. Although the effect of the identified variant on OX40L expression has not been fully identified, we know that both OX40L and OX40 are found at higher levels in patients with SLE, particularly those with more active disease and with a more severe disease phenotype (as indicated by the presence of nephritis) 55. These findings suggest that these proteins contribute to the increased number and more active phenotype of memory B cells in SLE.

GWAS data sets also show disease association with many of the genes associated with the development of immunologic memory including *BACH2*,* PRDM1*, and *IKZF3* 13, 14, 56 (Table 1 and steps 6–8 in Figure 2). The exact effect of the risk variant in each of these genes on function is not yet known, but these associations hint that the proteins may contribute to the dysregulation of the developmental pathways in memory and long‐lived plasma cells. Of particular interest, we know that mice lacking Aiolos fail to develop immunologic memory although they have normal initial responses to antigen exposure and are at increased risk of developing B cell lymphoma 57. Aiolos^–/–^ mice also develop a lupus‐like disease in old age, with typical autoantibodies and immune complex deposition 57. This autoimmune phenotype comes about as a result of a B cell defect with retention of both relatively normal T cells and innate immune system 57.

Two polymorphisms in *BANK1* have been identified as associated with risk 13, 14, 40, 58 (Table 1). Both impair BCR and CD40 signaling and are associated with an expansion of memory B cells in vivo 58 (step 4 in Figure 2). *BANK1* encodes a scaffolding protein that acts during BCR signaling by promoting phosphorylation of Src tyrosine kinases such as Lyn through enabling protein–protein interactions between them and inositol 1,4,5‐trisphosphate receptor 59. One of the risk polymorphisms (rs10516487) is associated with alternative splicing which results in a protein that forms larger and more widespread scaffolds within the cytoplasm 59. The effect of the other risk variant (rs17266594) on BANK‐1 expression is currently unknown.

## Extrinsic factors

Failure of tolerance in SLE is due in large part to the intrinsic defects discussed above, but it is important to note that B cells develop in an abnormal milieu in lupus patients compared to healthy controls. Analysis of bone marrow aspirates from patients with SLE demonstrates some interesting findings 60. First, there were more apoptotic cells within lupus bone marrow, potentially exposing developing pre–B cells to more nuclear self antigens and propagating the development of autoreactive BCRs 60. Second, there were increased numbers of CD4+ T cells, macrophages, and plasma cells, creating a proinflammatory environment 60. These inflammatory cells produced significant amounts of IFN (a high level of the cytokine in the bone marrow was associated with increased disease activity), which has profound effects on B cell development 61. An abundance of IFN in bone marrow leads to arrested development at the early stages and expansion at the transitional B cell stage 61. Similarly, the lymphoid tissue in SLE is also altered 62. The GCs are areas with a high proportion of cells undergoing proliferation and apoptosis. Under normal circumstances, apoptotic cells are rapidly cleared, but in SLE there are well‐known defects in this process (for extensive review, see ref. 63) potentially leading to persistence of apoptotic material including nuclear components and perpetuating the antigen‐driven B cell response 62.

Although epigenetic annotation of risk variants identifies the B cell as the main cell of interest for the disease, risk variants affecting many aspects of the immune system from innate to adaptive have been described 14, 64, 65, 66. Many of the risk loci associated with SLE discussed in this review also affect genes with multilineage functions. For example, the *IKZF1* variant has also been shown to affect expression of complement components and several IFN response genes, while Lyp is required for T cell receptor signaling 14, 67.

Another good example of this is the variant affecting *PRDM1*, which encodes for B lymphocyte–induced maturation protein 1 (BLIMP‐1), a transcription factor crucial for plasma cell differentiation. The effect of the risk variant rs548324, however, is demonstrable in dendritic cells (DCs), which show a reduction in expression as a result of the variant, a reduction not replicated in B cells 68. BLIMP‐1 knockout in DCs is associated with an increase in IL‐6 secretion as well as increased numbers of follicular T cells and GC B cells, with female mice developing SLE‐like disease 68. Therefore, while the ultimate phenotypic outcome of a specific variant may be B cell dysfunction, the actual molecular abnormality may not be B cell specific.

## Summary

Although the immunologic abnormalities in SLE are complex, numerous, and not fully understood, B cells have a central role in the development of the disease, from driving immune complex production to secretion of proinflammatory cytokines. Since SLE is an archetypal complex disease, the genetic association data reflect that complexity, with many variants, affecting several areas of the immune system 13, 14.

The advent of GWAS has identified many potential abnormal pathways relevant to the breaking of self tolerance in SLE (summarized in Table 1). The data emphasize the central role of B cells, with a significant proportion of identified genes affecting B cell function and epigenetic annotation reinforcing this 13, 14, 65, 66. GWAS have also helped to explain clinical variability between individuals—it is easy to see how each patient is likely to have a different combination of risk variants resulting in a pattern of immune dysfunction unique to that individual. We can already see how variants in TLR genes can help predict disease and serologic markers, or how variants in the gene for Lyn can protect against hematologic involvement 36, 49.

Traditionally, B cell–targeted therapy has mostly been focused on depletion of numbers (e.g., through targeting CD20‐mediated depletion through rituximab), but with our growing understanding, less crude approaches are being investigated. For example, Aiolos depletion therapy with the CC‐220 molecule results in down‐regulation of genes mediating B cell differentiation, reduces proliferation, and inhibits antibody secretion in B cells from SLE patients 69. Emerging therapy such as epratuzumab (a monoclonal antibody to CD22) has been shown to block B cell differentiation and activation directly through selective inhibition of BLIMP‐1 (encoded by *PRDM1*), demonstrating how data from GWAS can inform future drug therapy 70.

Although the results from GWAS confirm the complexity of SLE pathogenesis and show that different intrinsic B cell abnormalities are likely to drive disease in each individual case, the principles underlying the loss of central tolerance (deficient BCR signaling) and peripheral tolerance (dysregulated T cell–B cell interaction) are universal. Genetic information helps us to personalize therapy, whereby relevant molecules can be targeted through understanding the exact pathways involved in an individual's disease.

## Author Contributions

Drs. Karrar and Cunninghame Graham drafted the article, revised it critically for important intellectual content, and approved the final version to be published.
